# Short-Term Changes in Anemia and Malaria Parasite Prevalence in Children under 5 Years during One Year of Repeated Cross-Sectional Surveys in Rural Malawi

**DOI:** 10.4269/ajtmh.17-0335

**Published:** 2017-08-07

**Authors:** Alinune N. Kabaghe, Michael G. Chipeta, Dianne J. Terlouw, Robert S. McCann, Michèle van Vugt, Martin P. Grobusch, Willem Takken, Kamija S. Phiri

**Affiliations:** 1 Center of Tropical Medicine and Travel Medicine, Department of Infectious Diseases, Academic Medical Center, University of Amsterdam, Amsterdam, The Netherlands;; 2 School of Public Health and Family Medicine, College of Medicine, University of Malawi, Blantyre, Malawi;; 3 Lancaster University, Lancaster Medical School, Lancaster, United Kingdom;; 4 Malawi-Liverpool Wellcome Trust Clinical Research Program, Queen Elizabeth Central Hospital, College of Medicine, Blantyre, Malawi;; 5 Liverpool School of Tropical Medicine, Liverpool, United Kingdom;; 6 Laboratory of Entomology, Wageningen University and Research Centre, Wageningen, The Netherlands

## Abstract

In stable transmission areas, malaria is the leading cause of anemia in children. Anemia in children is proposed as an added sensitive indicator for community changes in malaria prevalence. We report short-term temporal variations of malaria and anemia prevalence in rural Malawian children. Data from five repeated cross-sectional surveys conducted over 1 year in rural communities in Chikwawa District, Malawi, were analyzed. Different households were sampled per survey; all children, 6–59 months, in sampled household were tested for malaria parasitemia and hemoglobin levels using malaria rapid diagnostic tests (mRDT) and Hemocue 301, respectively. Malaria symptoms, recent treatment (2 weeks) for malaria, anthropometric measurements, and sociodemographic details were recorded. In total, 894 children were included from 1,377 households. The prevalences of mRDT positive and anemia (Hb < 11 g/dL) were 33.8% and 58.7%, respectively. Temporal trends in anemia and parasite prevalence varied differently. Overall, unadjusted and adjusted relative risks of anemia in mRDT-positive children were 1.31 (95% CI: 1.09–1.57) and 1.36 (1.13–1.63), respectively. Changes in anemia prevalence differed with short-term changes in malaria prevalence, although malaria is an important factor in anemia.

## INTRODUCTION

*Plasmodium falciparum* malaria infection is a major contributing factor to anemia in African children, with both conditions responsible for high morbidity and mortality.^1^ Children below 5 years bear the highest proportion of anemia worldwide.^2^ Although malaria accounts for most of the anemia in African children in high transmission settings,^2,3^ malnutrition (macronutrient deficiency), human immunodeficiency virus (HIV), and helminthic infections also play roles.^4,5^

Malaria control interventions are intended to reduce malaria-related morbidity and mortality, which is challenging to define and measure in sub-Saharan Africa.^6,7^ An inverse relationship between malaria parasite density and hemoglobin level exists in children—increasing parasite density leads to lower hemoglobin levels and increasing anemia prevalence.^8,9^ Higher parasite densities are seen in high- than in low-transmission settings.^10–12^ Reduction in malaria burden, owed to the scale-up of control efforts, reduces anemia prevalence in children.^3,13^ In stable malaria transmission areas, anemia prevalence/hemoglobin level has been suggested as an additional impact indicator for changes in malaria prevalence.^3,6,14^ Anemia prevalence is reported to be more sensitive to changes in malaria burden compared with parasite prevalence in long-term surveys.^6,15^ High-transmission settings are associated with low parasite density and high hemoglobin levels. As hemoglobin assessment for anemia is affordable and reliable in field surveys, anemia may be a suitable indicator of changes in malaria burden in community surveys.

Short-term variations in community malaria and anemia prevalence during different malaria exposure seasons have not been adequately explored in repeated cross-sectional studies. These variations reflect the potential to use anemia prevalence as an indicator of malaria burden in a community. We analyzed data from one year of repeated cross-sectional household surveys^16^ to describe trends and associations of anemia and parasitemia in children 6- to 59-months old and discuss whether anemia prevalence can be used as an indicator of malaria prevalence.

## METHODS

### Study design and setting.

The data analyzed were from a rolling malaria indicator survey (rMIS)^16^ conducted in Chikwawa district in Southern Malawi. rMIS involves repeating cross-sectional household surveys within a defined area to measure malaria indicators.^17^ In this case, the indicators included malaria and anemia in children aged 6–59 months.^16^ The objective of the primary study was to implement rMIS as a household malaria survey at subdistrict level and provide continuous and readily available estimates of malaria and anemia prevalence. rMIS was also implemented to identify hotspots. The study setting has been previously described.^16^ Briefly, the primary study was conducted in the perimeter of the Majete Wildlife Reserve (MWR) a rural part of Chikwawa district from April 2015 to April 2016. The rural villages in MWR perimeter are grouped into community-based organisations (CBO), averaging eight villages. Each village has a population ranging from 58 to 1,500 people. rMIS was conducted in seven CBOs, termed “focal areas A, B, and C,” with a total of 61 villages, approximately 6,600 households, and 24,500 people. The population around the reserve relies mainly on rain fed subsistence and small-scale commercial crop farming for their livelihood. Transmission of (mainly *P. falciparum*) malaria occurs throughout the year, peaking during and after the rainy season (December to May). The rainy season is associated with flooding and abundant mosquito larval habitats. The main malaria vectors are *Anopheles gambiae* s.s., *Anopheles arabiensis*, and *Anopheles funestus*.^18^

### Participants.

Survey participants were children aged 6–59 months living in the MWR perimeter. Children were eligible for inclusion in the survey if 1) they slept in a household selected for rMIS the previous night; 2) they were a permanent member of the household; 3) the head of the sampled household or a legal guardian consented to their participation; and 4) they had no signs of severe illness. Children with signs of severe illness, defined as impaired consciousness, multiple convulsions, prostrations, deep breathing, circulatory collapse, clinical jaundice, and spontaneous bleeding, were urgently referred to the nearest health facility.

### Data collection.

A nurse and two to four research assistants interviewed the guardians of invited children using a tablet-based questionnaire; data were collected and managed using openHDS. The questionnaire and blood testing (with exclusion of malaria smears) were adapted from standardized Malaria Indicator Survey (MIS) (http://www.malariasurveys.org/toolkit.cfm). The questionnaire recorded malaria symptoms (fever or history of fever in preceding 48 hours), and recent treatment (within 2 weeks) for malaria. Treatment details for fever were confirmed from the participant’s health record passport. Blood tests included a malaria parasite test using a malaria rapid diagnostic test (mRDT) (*SD Bioline malaria Ag Pf* HRP-2; Standard Diagnostics Inc, Korea) for all participants and hemoglobin level using Hemocue 301^®^ (Hemocue, Angelholm, Sweden). *SD Bioline malaria Ag Pf* meets the World Health Organisation (WHO) procurement criteria; it has 95% and 99% panel detection scores at low (200 parasites/μL) and high (2,000 or 5,000 parasites/μL) densities, respectively, in clinical samples.^19^

Height, weight, and mid-upper arm circumference were measured using a height board, calibrated analogue weighing-scale, and mid-upper-arm circumference tape, respectively; all accurate to the nearest one decimal place. A digital electronic thermometer was used to measure axillary temperature (to nearest one decimal place). Fever was temperature 37.5°C and above.

Adult mosquitoes were sampled using a Suna trap^20^ over two consecutive nights per house. The Suna trap was set indoors one night and outdoors the other night, with the order determined by a coin flip. All *Anopheles* mosquitoes were separated by sex and identified morphologically according to Gillies and Coetzee.^21^

Hourly temperature and rainfall were recorded using a HOBO^®^ weather stations (Onset Computer Corporation, Massachusetts) in focal area B; temperature was summarized as the daily mean and rainfall as the daily total.

### Sample size and sampling.

A sample size of 300 households per round (100 per focal area) was initially calculated based on a planned 12-sampling rounds within one year to exhaust all households in an entire focal area. The sample size was later adjusted, after considering logistical capacity, to 270 households (90 per focal area) every 3 months. Adult mosquitos were collected in 3/4 of the rMIS households (67 households); this proportion was based on the logistical capacity of the research team to efficiently set the traps in the evening and collect the mosquitoes in the morning on two consecutive days.

A household mapping of the study site was conducted between August and November 2014. The sampling process for households has been described elsewhere.^16,22^ Briefly, based on the household locations, we used simple inhibitory sampling, a randomized probability sampling with spatial regularity constraint technique, in the first two rounds. Three subsequent samples were based on an adaptive geostatistical design (AGD) sampling.^16,22^ AGD sampling surveys used prediction results from preceding rMIS rounds to sample new locations of high estimate uncertainty for the subsequent rounds. The estimate used was malaria prevalence. The proportion of households for adult mosquito sampling was selected from rMIS households using random sampling.

### Risk of bias.

The initial sample in AGD is a probability-based sample, albeit restricted to induce a degree of spatial regularity into sampled locations, and increasing efficiency without a risk of subjective bias.^22^ Each household that meets the AGD constraints is equally likely to be sampled, hence, a nonzero probability of being sampled. Entomology households were also randomly sampled from the rMIS households.

### Variables.

Anemia was defined as mild, moderate, and severe using hemoglobin level cutoffs of 10–10.9 g/dL, 7.0–9.9 g/dL, and less than 7.0 g/dL, respectively. Predictor variables were mRDT result, sex, age, nutritional status, and ownership of insecticide-treated nets (ITNs). mRDT results were classified as mRDT positive or negative. mRDT-positive results were categorized “treated” (had taken antimalarial drugs within the preceding 2 weeks or currently taking AMDs) or “untreated” (not recently treated). Untreated mRDT positives were either symptomatic or asymptomatic based on the presence/absence of 1) fever and/or 2) reported malaria symptoms during the survey.

### Statistical methods.

We used R statistical package, (version 3.3.1)^23^ for statistical analysis. Z-scores of weight-for-height, weight-for-age, and height-for-age were calculated using the WHO anthro R scripts (http://www.who.int/childgrowth/software/en/). Univariate and multivariate analyses of predictors for anemia and hemoglobin were also calculated. We used a modified Poisson regression model^24^ to determine adjusted relative risks of anemia for mRDT-positive children (because the outcome was not rare). Predictors for anemia were chosen *apriori*.

### Ethical considerations.

Ethical review and approval were provided by the College of Medicine Review and Ethics Committee in Malawi (P.09/14/1631). An informed consent was administered to the head of household or household member above 18-years old in Chichewa (the local language). Children with mRDT-positive results or anemia were managed according to national guidelines or referred to a health facility for further treatment.

## RESULTS

### Summary of sampling rounds and participant characteristics.

Within a 12-month study period, a total of five data collection rounds were completed; data collection duration ranged from 1 to 2 months. From 1,568 sampled households, 1,377 were completed, and 894 children were enrolled during the five rounds. There were 323 (36.1%) children’s households which reported owning at least one ITN (Table 1). Prevalence of stunting (HAZ score < −2) was higher than both underweight (WAZ score < −2) and wasting (WHZ scores < −2). Anemia prevalence was 58.7% and significantly associated with age and mRDT result. Sex, ITN ownership, and nutritional status were not associated with anemia. Overall, the mean hemoglobin level was 10.5 g/dL (95% CI: 10.3–10.7 g/dL).

**Table 1:** Summary statistics for participants and prevalence of anemia (Hb < 11 g/dL)

Characteristic	Factor	Total (%)	Anemia *n* (%)	*P* value
Total number of children	–	894	525 (58.7)	–
Sex	Male	452 (50.6)	254 (48.4)	0.120
	Female	442 (49.4)	271 (51.6)	
Age category in months	6–11.9	100 (11.2)	84 (84.0)	< 0.001
	12–23.9	201 (22.5)	129 (64.5)	
	24–35.9	230 (25.7)	136 (59.1)	
	36–47.9	174 (19.4)	97 (55.6)	
	48–59.9	190 (21.2)	79 (41.6)	
Household owns at least one ITN (%)*	Yes	323 (36.1)	191 (59.1)	0.819
	No	569 (63.6)	332 (58.4)	
WHZ*	< −2 SD	182 (20.4)	107 (58.8)	0.964
	≥ −2 SD	703 (78.6)	412 (58.6)	
HAZ*	< −2 SD	394 (44.1)	242 (61.4)	0.139
	≥ −2 SD	499 (55.8)	282 (56.5)	
mRDT	Positive	302 (33.8)	223 (73.8)	< 0.001
	Negative	592 (66.2)	302 (57.0)	
Severe anemia	–	37 (4.1)	–	–
	Mean	95% confidence interval	–	–
Hemoglobin in g/dL	10.5	10.3–10.7	–	–

HAZ = height-for-age z score; Hb = hemoglobin; ITN = insecticide-treated bed net; mRDT = malaria rapid diagnostic test; SD = standard deviation; WHZ = weight-for-age z score.

* Missing data for some children. The following were denominators: household owns at least one ITN (N = 892); WHZ (N = 885); HAZ (N = 893).

### mRDT results and malaria symptoms.

**Figure 1.**
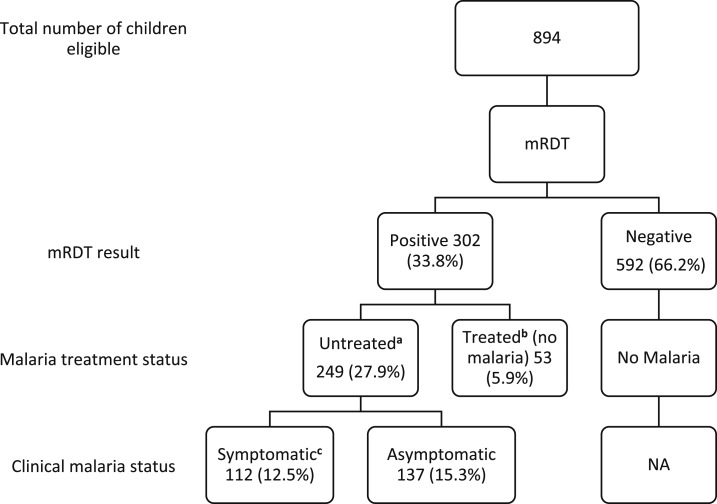
Symptomatic and asymptomatic malaria. The flow diagram shows symptomatic and asymptomatic mRDT-positive patients based on the presence/absence of malaria symptoms and recent malaria treatment. ^a^ mRDT positive without prior malaria treatment in past 2 weeks; ^b^ mRDT positive from presumed residual antigens in blood and not active infection, based on antimalaria treatment in previous 2 weeks; ^c^ Malaria symptoms during the survey were either one or both of the following 1) confirmed fever (axillary temperature of 37.5°C or above) and 2) reported fever within the past 48 hours. mRDT = malaria rapid diagnostic test.

The total number of mRDT-positive children was 302 (33.8%), who mostly (27.9%) were untreated mRDT positive (Figure 1). Asymptomatic parasitemia prevalence was 15.3% in the children.

### Mean hemoglobin level of children by malaria status.

**Figure 2.**
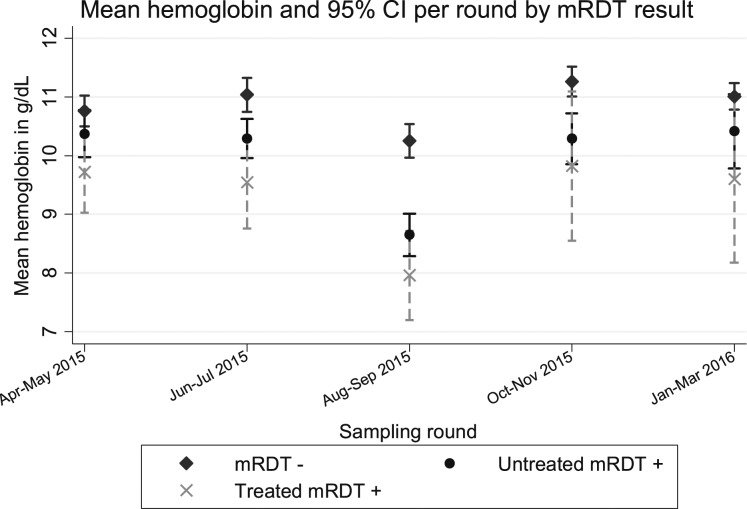
Graph for mean hemoglobin (mg/dL) by mRDT category. Children with negative mRDT results had higher hemoglobin level compared with those with an mRDT-positive result. mRDT = malaria rapid diagnostic test; CI = confidence interval.

Overall, the mean hemoglobin level in mRDT-negative children, untreated and treated mRDT positives were 10.89 g/dL (95% CI: 10.78–11.00), 9.91 g/dL (9.68–10.13), and 9.21 g/dL (8.74–9.68), respectively. Figure 2 shows the variation in mean hemoglobin level between the three groups per sampling round.

### Distribution of malaria and anemia by age categories.

**Figure 3.**
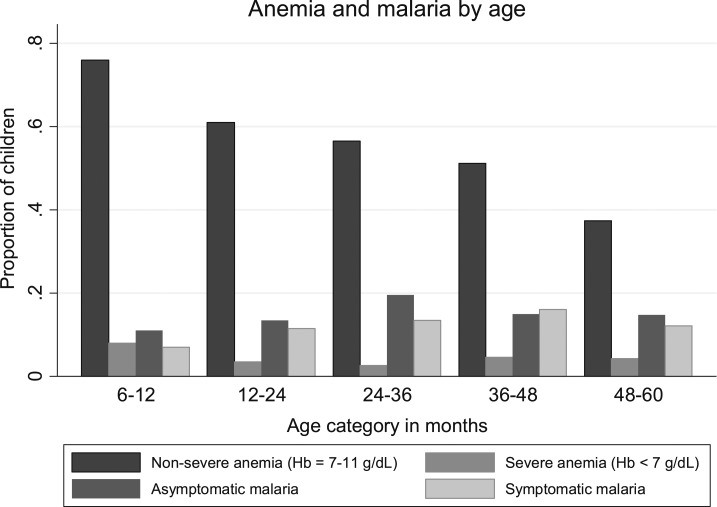
Proportion of parasitemic and anemic children by age group.

Over 80% of children aged 6- to 12-months old had any anemia. Anemia prevalence was highest in this category and gradually declined with age (Figure 3). Severe anemia prevalence was relatively low although highest in the youngest age category. The proportion of children with both symptomatic and asymptomatic parasitemia gradually increased with age from the youngest to 36- to 47.9-month categories.

### Prevalence of anemia, malaria parasitemia, and malnutrition by sampling rounds.

There were variations in temperature and rainfall during data collection, from the dry cooler season, in rounds 2 and 3, to the wet warmer season in rounds 4 and 5, respectively (Table 2). The highest prevalence of anemia and severe anemia were 73.7% and 15.4%, respectively, both in round 3. Up to 25% of the children had asymptomatic parasitemia in round 3. Mean female *Anopheles* mosquito density, females per house per night, was generally low; density was highest in round 2 and lowest in round 5. Overall, 247 (27.6%), 195 (21.8%) and 95 (10.5%) children were underweight, stunted, or wasted (*z*-score < −2 standard deviations), respectively.

**Table 2:** Rainfall, temperature, anopheline density and prevalence of anemia, malaria parasitemia and malnutrition in children < 5 years by sampling round

Year	2015								2016		
Months	Apr	May	Jun	Jul	Aug	Sep	Oct	Nov	Jan	Feb	Mar
Sampling rounds	1 (*N* = 185)		2 (*N* = 182)		3 (*N* = 175)		4 (*N* = 171)		5 (*N* = 181)		
Average temperature in degrees Celsius	22.3		20.8		24.5		30.1		28.4		
Total rainfall in mm	12.0		0		0		17.0		57.2		
Mean anopheles mosquito density*: (95% CI)	0.13 (0.04–0.23)		0.27 (0.19–0.35)		0.17 (0.10–0.24)		0.11 (0.04–0.19)		0.07 (0.03–0.10)		
Any anemia – Hb < 11 g/dL; % (95% CI)	58.9 (52–66)		58.2 (51–65)		73.7 (67–80)		48.0 (40– 55)		54.7 (47–62)		
Severe anemia – Hb < 7 g/dL; % (95% CI)	3.2 (1.5–7.1)		2.1 (0.8–5.7)		15.4 (10.7–21.5)		0		0		
Total mRDT positive; % (95% CI)	36.8 (29.8–43.7)		47.3 (40.0–54.5)		43.4 (36.1–50.8)		28.0 (21.3–34.8)		13.3 (8.3–18.2)		
Untreated mRDT positive; % (95% CI)	27.6 (21.1–34.0)		40.1 (32.8–47.0)		35.4 (28.3–42.5)		25.1 (18.6–31.7)		11.0 (6.5–15.6)		
Asymptomatic untreated mRDT positive; % (95% CI)	10.3 (5.9–14.6)		17.5 (12.0–23.0)		25.1 (18.7–31.6)		17.5 (11.8–23.2)		6.6 (3.0–10.3)		
HAZ < −2; *n* (%)†	41 (22.2)		44 (24.2)		47 (26.9)		40 (23.4)		23 (12.7)		
WHZ < −2; *n* (%)†	20 (10.8)		20 (11.0)		11 (6.3)		23 (13.5)		21 (11.6)		

HAZ = height-for-age z-score (stunting); WHZ = weight-for-height z-score (wasting); mRDT = malaria rapid diagnostic test.

* Total *Anopheles* females collected per house per night regardless of whether sampling was done indoors or outdoors.

† Nine children and one child had incomplete data for WHZ and HAZ, respectively, and were excluded from the denominator.

**Figure 4.**
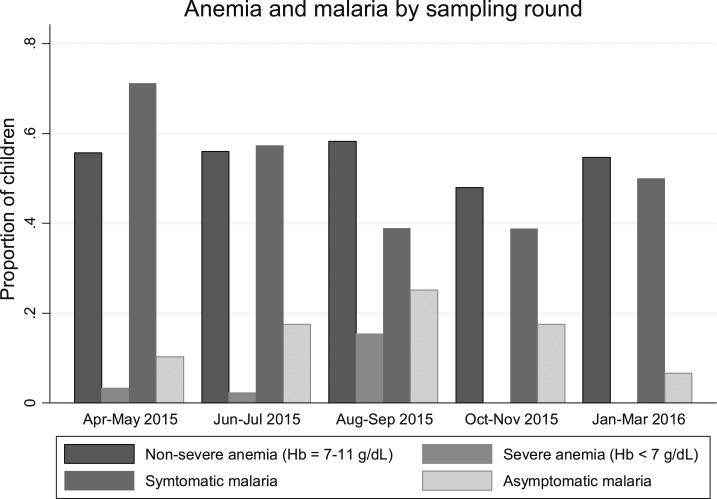
Anemia and parasitemia by sampling round.

There is an obvious trend in asymptomatic malaria prevalence among the five rounds: an increase between round 1 and 3 followed by a steady decrease from round 3 to 5 (Figure 4). The variations in anemia and severe anemia prevalence over the five rounds lack an obvious trend; anemia remains above 50% in rounds 1 and 2, peaks after highest malaria prevalence and mosquito density (Table 2) then declines and rises in rounds 4 and 5, respectively. Anemia prevalence remains high even after decrease in malaria parasite prevalence between round 4 and 5.

### Crude and adjusted risk ratios of anemia from malaria.

Overall, mRDT-positive children had a higher risk of anemia compared with mRDT negatives in both the unadjusted and adjusted (Table 3). There was no difference statistically significant in anemia risk between mRDT-positive and -negative children by sampling round. After adjusting for age, wasting, and stunting, children with an mRDT-positive result had overall relative risk of 1.36 (95% CI: 1.13–1.63). Increasing age, sampling round, and focal area increased hemoglobin level (Supplemental Table 1). mRDT-positive result and stunting decreased the hemoglobin level.

**Table 3:** The unadjusted and adjusted relative risks of anemia in children with mRDT-positive result by sampling round

Round	1	2	3	4	5	Overall
Unadjusted relative risk (95% CI)	1.19 (0.78–1.77)	1.28 (0.87–1.88)	1.32 (0.92–1.87)	1.28 (0.78–2.04)	1.33 (0.72–2.27)	1.31 (1.09–1.57)
Adjusted* relative risk (95% CI)	1.31 (0.84–2.02)	1.29 (0.87–1.89)	1.31 (0.91–1.88)	1.52 (0.90–2.54)	1.31 (0.71–2.26)	1.36 (1.13–1.63)

* Adjusted for age, weight-for-height, and height-for-age.

## DISCUSSION

By conducting epidemiological and entomological surveys every 2–3 months, we describe short-term variations in malaria parasite and anemia prevalence in one year of data collection. We used small teams of research personnel and easy-to-use diagnostic tools to conduct the survey, making it feasible for monitoring community burden as well as active case detection and treatment. Using AGD sampling, the survey efficiently measured spatio-temporal heterogeneity in the prevalence of malaria parasites.^16^

Overall, the adjusted and unadjusted relative risks provide evidence that malaria parasitemia is associated with anemia in children. These findings are similar to studies in sub-Saharan Africa^8,25^ and, although from a cross-sectional study design, support the role of malaria infection in anemia in children.^26^ Malarial anemia results from loss of both infected and uninfected erythrocytes, dyserythropoiesis, and suppressed erythropoiesis.^26–28^

Trends in anemia and malaria parasite prevalence in the survey suggest that anemia prevalence in children does not indicate short-term seasonal changes in malaria parasite prevalence. In previous studies, decrease in malaria burden was associated with a reduction in anemia prevalence. These studies had longer follow up time (one to several years between surveys), fewer survey points (2 transmission points), or conducted before and after an intervention.^6,15,29–31^ In the current study, data were collected frequently (every 2–3 months) over 1 year, allowing the prevalence to be measured in more seasons and at a finer scale than previously; anemia prevalence in our findings lacks an obvious trend with respect to season or parasitemia. Anemia is still an important indicator for malaria morbidity. Long-term maintenance of low malaria burden, through scaling up of interventions, is required for population effects (e.g., reduced anemia prevalence).

Older children had more malaria parasitemia compared with younger children. Studies have shown that older children are less likely to sleep in ITNs and more likely to use older, less effective, and worn out ITNs compared with pregnant women, infants, or younger children.^32–35^ ITN ownership was low in the study; households may prioritize younger siblings to use better quality ITNs, leaving older ones less protected. Higher use and better quality of ITNs in younger age groups also relates to free access by the mothers during antenatal care or during the child’s vaccine; in addition, higher ITN and better quality ITN usage may result from younger children sharing sleeping space with their parents.^34^

Anemia prevalence was comparatively higher in younger than in older children, similar to other studies.^36,37^ In this study, anemia in children aged 6- to 11.9-months old was exceptionally high. A possible cause of anemia in younger children is iron deficiency resulting from high iron demand for rapid growth, low iron intake from low iron concentration in breast milk and weaning food, and frequent infections affecting feeding.^38,39^ There was evidence of chronic malnutrition in the study population from the high prevalence of stunting. Stunting was associated with a decrease in hemoglobin level in the multivariate analysis. The communities relied mainly on rain-fed crop farming (for their diet) which may not consistently yield adequate harvest and has low iron content. Also, children in endemic regions acquire immunity to malaria as they grow older from repeated parasite exposure; this immunity also reduces the effect of malaria parasitemia on hemoglobin level by maintaining low parasite densities.^40^

Hemoglobin was lower in treated mRDT-positive children compared with untreated mRDT-positive children. Studies have demonstrated an initial sharp decrease in hemoglobin during malaria treatment with artemisinin-based combination therapy before a gradual rise to normal levels after treatment completion.^41–43^ This low hemoglobin level takes on average 10 days and supports the relatively low hemoglobin level found in children who had recently taken AMD. The use of mRDTs and information on recent treatment of malaria in the survey offers a new category for malaria exposure (i.e., *treated* mRDT positive). This category should be considered an important determinant of hemoglobin level and anemia in children in community surveys.

There was a high prevalence of undiagnosed and untreated anemia, parasitemia and malnutrition in the rural community. The high prevalence of asymptomatic malaria in some months of the year is especially important because this likely drives malaria transmission in subsequent months. A recent study in Tanzania^44^ has reported higher prevalence of gametocytes in asymptomatic than in symptomatic school-aged children, highlighting the role of asymptomatic parasitemia in malaria transmission. An overall anemia prevalence of 58.7% in randomly selected households indicates that anemia is a severe public health problem in the study population.^2^ A further 4.1% of children overall had hemoglobin less than 7 g/dL. The prevalence of stunting in children in the study area was higher than 2015–2016 Malawi Demographic Health Survey.^45^ Childhood stunting irreversibly affect adult height, school performance, and adult income.^46^

High burden of anemia, malaria, and malnutrition in rural communities calls for an integrated control approach.^47^ Complex relationships of the three conditions have been previously reported with both malaria and malnutrition being risk factors for anemia: malnutrition predisposing to malaria infection and worsening its severity, and commonly coexisting risk factors for all three conditions.^10,48,49^ A systematic review examining the correlation between malaria and macronutrient deficiency however reported that malaria is associated more with anemia in children than macronutrient deficiency.^5^

This study has limitations. mRDTs have low specificity and positive predictive value for active malaria infection in endemic settings as they detect parasite antigens which persist in the blood after treatment and clearing of parasite. By detecting cleared infections, mRDT can over-report malaria parasitemia, and therefore, caution should be taken when reporting parasite prevalence based on mRDT results. We report mRDT-positive children in separate categories: treated and untreated. Current mRDTs are incapable of determining parasite density and detecting low density parasitemia. The use of mRDTs in the survey simplified data collection and treatment in the communities. Because this was a secondary analysis, samples in each round were not powered to detect precise estimates of association between malaria and anemia.

We only used hemoglobin level measured by Hemocue^®^ to report anemia. Anemia mechanisms and common etiologies of anemia in children, such as soil-transmitted helminths (STH), schistosomiasis, parvovirus B19, and HIV, were not investigated in the study. Both STH and schistosomiasis have been previously reported to be high in this region although variation in prevalence with time has not been reported.^50–52^ Sickle cell and thalassemia were not tested; the objective of the primary study was to implement rMIS as an alternative household malaria survey using small district teams and field-based tests. However, two previous studies in the same region reported a sickle-Hb-gene frequency of 0.11^53^ and prevalence of sickle cell disease of 2%^4^ in 400 infants and 101 children aged 6- to 60-months old, respectively.

From the study findings, anemia was associated with malaria parasitemia although the short-term temporal trends between the two parameters were different. Hemoglobin level was affected by malaria treatment in the preceding 2 weeks; younger children were disproportionately affected more by anemia than older children. Rural communities have a high prevalence of undiagnosed and untreated anemia, parasitemia, and malnutrition and therefore require integrated control efforts.

## Supplementary Material

Supplemental Table.
