# Supplementary material for: Short-Term Changes in Anemia and Malaria Parasite Prevalence in Children under 5 Years during One Year of Repeated Cross-Sectional Surveys in Rural Malawi
Source: Am J Trop Med Hyg. 2017 Aug 7;97(5):1568–75. doi: 10.4269/ajtmh.17-0335 (PMC5817775; doi:10.4269/ajtmh.17-0335)
Supplement: Supplementary file 1 [file tpmd170335.SD1.pdf]

Supplemental Table 1

Estimates and 95% confidence interval for predictors of hemoglobin level

	Factor	Estimate	Lower confidence interval	Upper confidence interval
mRDT result	Negative	Ref	–	–
	Positive	−0.92103	−1.14422	−0.69785
Age category in months	6–12	Ref	–	–
	12–24	0.63563	0.27234	0.99893
	24–36	1.02236	0.66417	1.38055
	36–48	0.78234	0.40846	1.15622
	48–60	1.25521	0.88738	1.62304
Stunting	HAZ > −2	Ref	–	–
	HAZ < −2	−0.25948	−0.46048	−0.05847
Wasting	WHZ > −2	Ref	–	–
	WHZ < −2	−0.07593	−0.32339	0.17152
